# Supplementary material for: The portfolio effect in a small-scale fishery reduces catch and fishing income variability in a highly dynamic ecosystem
Source: PLoS One. 2022 Aug 5;17(8):e0271172. doi: 10.1371/journal.pone.0271172 (PMC9355173; doi:10.1371/journal.pone.0271172)
Supplement: Attachment — Submitted filename: Response to reviewers.pdf [file pone.0271172.s001.pdf]

Andrés Vargas
Assistant professor, Universidad del Norte
Barranquilla, Colombia

06/14/2022

Dear Editor,

We are very pleased to submit the new version of the paper "The portfolio effect in a small-scale fishery reduces catch and fishing income variability in a highly dynamic ecosystem". We want to thank the reviewers for their valuable comments. They helped us to improve the quality of the article.

In the next paragraphs, we will address their comments. Our responses are in red.

Reviewer #1

1. Introduction:

a) "specially if their capacity to smooth consumption through access to credit..." this is not very clear and can probably be reworded.

R/ It now reads

"…specially if their limited access to key markets, like credit and insurance, constrain their ability to maintain their consumption of goods and services through time"

b) When you speak about artisanal fisheries, you repeat what you have said in the previous section. Perhaps delete the first sentence of the second paragraph, and instead refer to "Strategies to recuse income risks among artisanal fisheries include....."

R/ Changed as suggested

c) do you really need the word "asynchronously" here and in the rest of the ms? Isn't it enough with just "fluctuate"?

R/ Yes, since the degree of species synchrony is key to the stability of the species community

d) please explain more clearly the links between ecosystem diversity and function. This is a long-standing debate with lots of opinions. You can elaborate and provide further references. This is a favorite from my perspective, but it is very old and probably lots of better and newer things out there (Elmqvist et al. 2003): https://esajournals.onlinelibrary.wiley.com/doi/pdf/10.1890/1540-9295(2003)001[0488:RDECAR]2.0.CO;2 and hopefully relevant to your study.

R/ Thanks for the suggestion. A brief explanation on the relationship between biodiversity and ecosystem function and services is added, including two references: Elmqvist et al. (2003) and Balvanera et al. (2015).

"The relationship between income variability and catch diversity stems from the role that biodiversity plays in the productivity and stability of natural systems [9, 10]. Three main mechanisms have been proposed to study the link between biodiversity and ecosystem function and services [11]: 1) complementary differences between species, 2) dominance by high-performing species, and 3) differential response of species to environmental conditions. The later of these, referred to as the insurance hypothesis, means that aggregate ecosystem properties vary less in more diverse communities [12]. For the well-being of society, the insurance hypothesis lends support to the claim that high diversity of response to environmental change among species is critical to the maintenance of valuable ecosystem services [13]".

e) on line 44-45 you refer to survival. Is that correct?

R/ Changed to success

f) the fishers in the study: do you have any data that illustrates your points of how diversity stabilize income, and that they do not have access to credit, etc (last section of introduction)?

R/ The assertion is based on our observations during fieldwork in the area. To better illustrate it, we added statistical data concerning poverty in the area, as well as evidence from a nearby fishing community showing their limited access to credit.

2. Materials and methods

a) please check that all figures are referred to properly. All references to figures are "Fig ??" in the pdf I have access to (maybe this is just a formatting issue).

R/ Checked

b) line 114 "this effect" - please clarify what this refers to - it is unclear.

R/ The paragraph was placed in the wrong place. It was deleted

c) Line 17, refers to "methods" but this is consuming since it is already part or the methods section. Consider revising to "Analytical method" or similar.

R/ Changed as suggested

d) Line 124 "the the" - delete one "the"

R/ Changed as suggested

I had a hard time understanding the SUR model (and they results from it), but maybe that is just because it is outside of my area of expertise.

R/ We reworded the paragraph below equation (8) to better explain the model.

Discussion

a) maybe explain the mean-variance trade off for the uninformed reader?

R/ An explanation was added as suggested

"Generally, the modern portfolio theory builds a framework for the selection of investment portfolios that maximize the return for a given level of risk, or one that attain a desired level of return at a minimum risk. To implement the method, investors need to calculate the returns, variances, and covariances of assets. Based on this information, they decide how much of their wealth is allocated to each asset."

b) please consider if you want to include this discussion about functional diversity etc., and if so, include more references.

R/ We decided not to include the discussion

References:

Some references have full first names (e.g., 1, 30, 31, 32) but most don't. Please check the formatting. Reference 28 is missing a year, and 29 is missing a capital letter for the first name "Andersson S." Check all formatting and consistency with PLOS one requirements.

R/ List of references checked and formatted in accordance with PLOS requirements
